# Quantitative Electroencephalography in Hemodialysis: Scoping Review and Translational Framework

**DOI:** 10.2196/94560

**Published:** 2026-07-31

**Authors:** Meng-Hsun Tsai, Cheng-Hsiung Chan

**Affiliations:** 1 Department of Mechanical Engineering, National Yunlin University of Science and Technology, No. 123, Section 3, University Road, Douliu, Yunlin, 64002, Taiwan, 886 5-534-2601 ext 4102, 886 5-532-1719

**Keywords:** hemodialysis, quantitative electroencephalography, digital biomarker, cerebral blood flow, intradialytic monitoring, cognitive impairment, signal processing, multimodal synchronization

## Abstract

**Background:**

Cognitive impairment and cerebral dysfunction are common among patients with end-stage kidney disease undergoing maintenance hemodialysis. Repeated intradialytic hemodynamic stress, including reductions in cerebral blood flow and episodes of intradialytic hypotension, has been associated with adverse neurological outcomes. Quantitative electroencephalography (qEEG) offers a noninvasive approach for continuous assessment of cerebral activity; however, its potential role in dialysis monitoring remains unclear because of methodological heterogeneity and implementation challenges.

**Objective:**

This scoping review aimed to synthesize current evidence regarding qEEG alterations across the hemodialysis cycle and to evaluate the technical requirements for translating qEEG-derived features into future dialysis-related cerebral monitoring systems.

**Methods:**

A structured literature search was conducted in PubMed/MEDLINE, Embase, and IEEE Xplore for studies published between January 2005 and February 2026. After removal of duplicates, 554 records were screened, and 70 full-text articles were assessed for eligibility. Sixty-two studies met the inclusion criteria and were included in the qualitative synthesis. Evidence was organized according to the 3 temporal domains of the hemodialysis cycle: predialysis baseline, intradialytic exposure, and postdialysis recovery. In addition to clinical findings, engineering-related evidence concerning electroencephalography hardware, electrode systems, signal processing pipelines, artifact mitigation, multimodal synchronization, edge computing, and digital biomarker validation was reviewed.

**Results:**

Patients receiving maintenance hemodialysis consistently demonstrated baseline spectral slowing characterized by increased delta and theta activity, and reduced alpha power. During dialysis, multimodal imaging studies reported cerebral blood flow reductions of approximately 10% to 15%, accompanied by dynamic qEEG changes, including alterations in the alpha-delta ratio and slow wave activity. Connectivity and complexity measures provided complementary information regarding network-level and recovery-related responses but showed substantial variability across acquisition conditions and analytical pipelines. Major implementation challenges included electrical interference, motion artifacts, electrode instability, asynchronous physiological data streams, and limited standardization of preprocessing methods. Comparative analysis indicated that acquisition quality, artifact rejection performance, synchronization accuracy, and individualized baseline modeling are critical determinants of translational feasibility.

**Conclusions:**

Current evidence indicates that qEEG-derived measures have been reported in association with both chronic and intradialytic neurophysiological changes in patients undergoing hemodialysis. However, available evidence remains heterogeneous and insufficient to support routine clinical deployment. Future implementation will require standardized acquisition protocols, robust artifact mitigation, synchronized multimodal monitoring, and prospective validation studies. At present, qEEG-derived features should be regarded as candidate digital biomarkers and components of a proposed translational monitoring framework rather than clinically validated monitoring tools.

## Introduction

### Clinical Need for Cerebral Monitoring During Hemodialysis

Hemodialysis has substantially improved survival among patients with end-stage kidney disease. Despite these advances, neurological complications remain common and continue to affect quality of life and long-term outcomes. Cognitive impairment, executive dysfunction, and attention deficits occur more frequently in maintenance hemodialysis populations than in age-matched controls [1-4]. Although prevalence estimates vary among studies, accumulating evidence suggests that cerebral dysfunction represents a major but an undermonitored complication of chronic dialysis.

Several mechanisms have been proposed to explain these neurological abnormalities. Repeated intradialytic hemodynamic stress, fluctuations in blood pressure, osmotic shifts, and reductions in cerebral blood flow (CBF) may contribute to cumulative cerebral injury. Neuroimaging studies have demonstrated transient reductions in regional and global CBF during ultrafiltration, often accompanied by cognitive deterioration and white-matter abnormalities [5-8]. Intradialytic hypotension (IDH) and dialysis disequilibrium syndrome further increase the risk of cerebral stress [9,10]. However, routine monitoring in dialysis units remains largely limited to systemic physiological variables, such as blood pressure, heart rate, fluid balance, and dialysis adequacy. Direct assessment of cerebral function is rarely incorporated into clinical workflows.

Electroencephalography (EEG) provides a noninvasive method for continuously monitoring cortical activity with millisecond temporal resolution. Classical EEG studies in uremic encephalopathy reported generalized slowing of background rhythms [11,12]. More recent quantitative EEG (qEEG) investigations have demonstrated increased delta and theta activity, reduced alpha power, altered connectivity patterns, and changes in signal complexity among patients receiving hemodialysis [13-16]. These findings suggest that cerebral electrophysiological activity is sensitive to both chronic metabolic burden and acute intradialytic perturbations.

The concept of the brain as a “missing vital sign” in dialysis has therefore attracted increasing attention. Rather than implying an established clinical standard, this concept highlights the discrepancy between measurable cerebral dysfunction and the absence of routine neurophysiological monitoring. Existing evidence indicates that cerebral responses during dialysis are dynamic and individualized, emphasizing the need for monitoring approaches capable of capturing the temporal neurophysiological variation.

### Engineering Foundations of qEEG-Based Digital Biomarkers

Recent advances in biomedical engineering have transformed physiological monitoring from simple signal acquisition into integrated digital biomarker systems. Within this framework, physiological signals are processed through standardized acquisition, preprocessing, feature extraction, and validation pipelines to generate interpretable biomarkers that reflect biological states or treatment responses [17-19].

qEEG is particularly attractive in this context because it provides multiple feature domains, including spectral power measures, connectivity metrics, entropy-based complexity indices, and temporal dynamics. These features may characterize cerebral responses to hemodynamic and metabolic stress during dialysis. However, substantial methodological variability exists across published studies, limiting direct comparison and reproducibility.

Differences in EEG hardware platforms, electrode technologies, channel configurations, sampling frequencies, and preprocessing procedures contribute to inconsistencies in reported findings. Wet-electrode systems generally provide superior signal quality but require longer setup times and periodic maintenance. Dry-electrode systems improve usability and patient comfort but may be more susceptible to impedance variability and motion-related artifacts [20,21]. Similarly, artifact rejection approaches—including independent component analysis, adaptive filtering, and wavelet-based denoising—vary considerably across studies and directly influence signal quality and feature reliability [22-25].

Translation of qEEG into dialysis environments introduces additional engineering challenges. Dialysis units contain multiple sources of electrical interference, prolonged acquisition periods, patient movement, and asynchronous physiological data streams. Reliable deployment therefore requires robust signal acquisition, effective artifact mitigation, multimodal synchronization, and reproducible analytical pipelines. Emerging edge computing architectures may support near–real-time processing by performing artifact rejection, feature extraction, and event detection close to the point of acquisition while reducing computational latency [26-28].

Digital biomarker development further requires staged evaluation, including technical verification, analytical validation, and clinical validation [17,18]. Although qEEG has demonstrated physiological sensitivity in multiple studies, evidence supporting reproducible clinical deployment remains limited. Consequently, qEEG-derived metrics should currently be regarded as candidate digital biomarkers requiring further validation rather than as established clinical monitoring tools.

### Objective and Scope of This Scoping Review

Growing evidence links hemodialysis-associated physiological stress to measurable neurophysiological alterations. Nevertheless, the translation of these observations into practical cerebral monitoring systems remains limited [1-8]. One reason is that clinical findings and engineering considerations are frequently discussed separately, resulting in an incomplete understanding of implementation requirements.

This scoping review was conducted to systematically map current evidence regarding qEEG in the hemodialysis setting. The review organizes findings according to the 3 phases of the dialysis cycle: predialysis baseline, intradialytic exposure, and postdialysis recovery. Beyond summarizing clinical observations, the review evaluates technical factors that influence deployment feasibility, including EEG hardware selection, electrode systems, signal processing pipelines, artifact rejection strategies, synchronization approaches, edge computing requirements, and digital biomarker validation frameworks.

The objective is not to establish qEEG as a clinically validated monitoring technology but to identify evidence gaps, methodological variability, and engineering requirements relevant to future implementation. Within this framework, qEEG-derived features are considered candidate physiological indicators whose clinical utility remains to be established through prospective validation studies and standardized acquisition methodologies.

**Figure 1.**
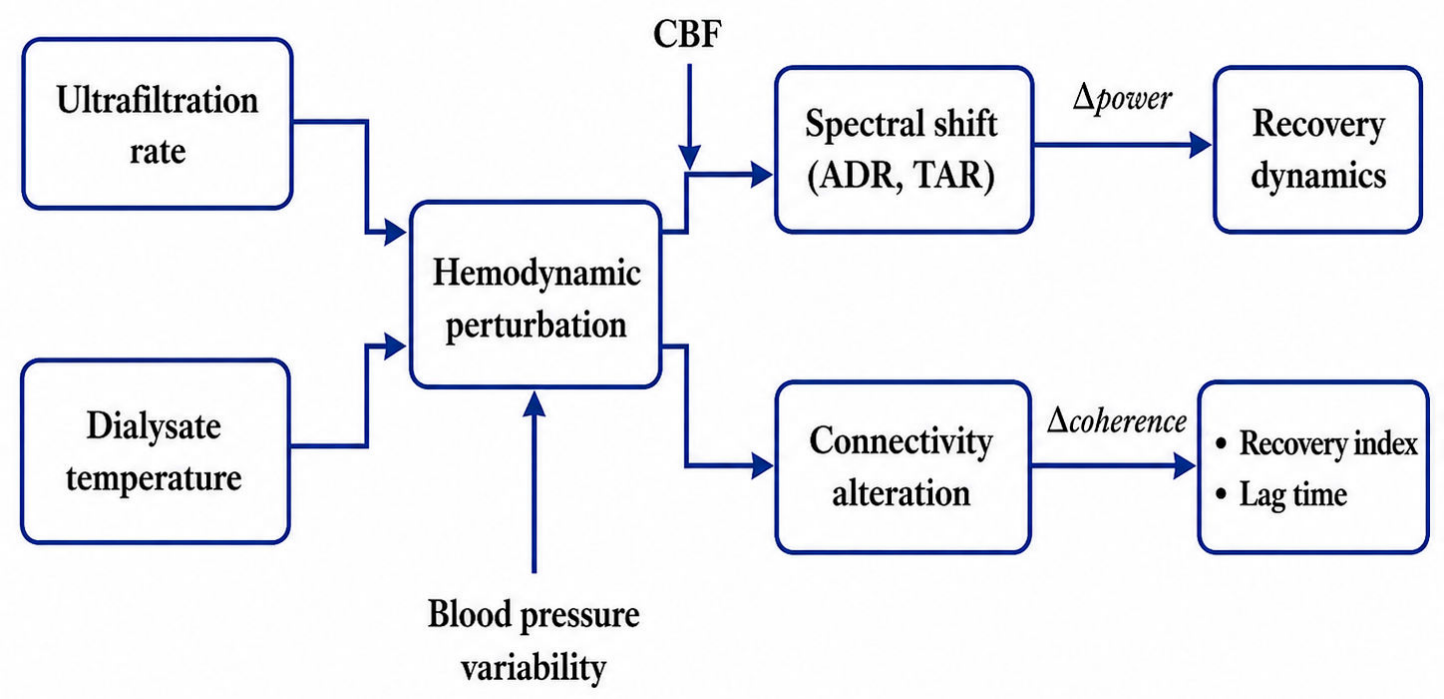
Conceptual dialysis-brain perturbation-response framework. ADR: alpha-delta ratio; CBF: cerebral blood flow; TAR: theta-alpha ratio.

The conceptual model adopted in this review views the hemodialysis session as a structured physiological perturbation. Treatment parameters, including ultrafiltration rate and dialysate conditions, act as controlled inputs; hemodynamic responses represent intermediate physiological states; and qEEG-derived features constitute measurable neural outputs. This perturbation–response perspective provides a practical framework for integrating clinical observations with engineering considerations and for guiding the future development of dialysis-related cerebral monitoring systems (Figure 1).

## Methods

### Study Design and Protocol Registration

This study was conducted as a scoping review to systematically map existing evidence regarding qEEG in the hemodialysis setting. The review was designed to identify, categorize, and synthesize heterogeneous evidence spanning clinical nephrology, neurophysiology, biomedical engineering, and digital health. Consistent with scoping review methodology, the objective was not to generate pooled effect estimates but to examine patterns, methodological variability, and translational considerations across the available literature [29-32]. The review was performed in accordance with the PRISMA-ScR (Preferred Reporting Items for Systematic Reviews and Meta-Analyses extension for Scoping Reviews) guidelines (Checklist 1) [29]. In addition, the review followed the methodological framework proposed by Arksey and O’Malley and subsequent guidance from the Joanna Briggs Institute for scoping reviews [30-32].

No prospectively registered or publicly accessible review protocol was available for this scoping review. The review question, information sources, eligibility criteria, screening approach, data charting domains, and synthesis strategy were specified during the planning stage and applied during the final screening and evidence mapping process. As the review was not registered in PROSPERO, OSF, or another public registry, deviations from a registered protocol could not be reported.

This review was conducted from a biomedical engineering and translational informatics perspective. Accordingly, the synthesis focused on EEG acquisition, signal processing workflows, artifact mitigation, multimodal synchronization, edge computing requirements, and digital biomarker validation, rather than on providing clinical nephrology practice recommendations.

### Information Sources and Search Strategy

A structured literature search was performed in PubMed/MEDLINE, Embase, and IEEE Xplore. These databases were selected to capture clinical, engineering, and digital health literature relevant to EEG- or qEEG-based cerebral monitoring in hemodialysis populations [17-19,29,30]. The search covered publications from January 2005 to February 2026.

Search terms were developed across three concept domains: (1) hemodialysis population, (2) EEG and qEEG, and (3) cerebral or cognitive outcomes. Controlled vocabulary and free-text terms were combined using Boolean operators. The complete database-specific search strategies for PubMed, Embase, and IEEE Xplore are provided in Multimedia Appendix 1 to ensure reproducibility [29,30]. No language restrictions were applied during the database search stage.

In addition to studies directly evaluating EEG or qEEG in hemodialysis populations, engineering-oriented publications addressing EEG acquisition systems, electrode technologies, artifact mitigation methods, signal processing pipelines, multimodal synchronization, wearable EEG platforms, edge computing architectures, and digital biomarker validation frameworks were also screened when relevant to translational implementation [18-28,33-35]. Additional contextual sources were identified through reference checking when they were directly relevant to the interpretation of dialysis-related cerebral physiology, neurophysiological monitoring, or implementation requirements.

### Eligibility Criteria and Evidence Roles

Eligibility criteria were defined according to the population, concept, and context of this scoping review. The population of interest was adult patients undergoing maintenance hemodialysis. The core concept was the use of EEG or qEEG to characterize neurophysiological alterations, cerebral dysfunction, or monitoring-relevant features. The context was the hemodialysis cycle, including predialysis baseline status, intradialytic exposure, and postdialysis recovery.

As the review aimed to synthesize both direct qEEG evidence and implementation-relevant supporting evidence, included sources were classified into 5 evidence roles. First, direct EEG or qEEG studies in adult hemodialysis populations were considered primary electrophysiological evidence if they reported EEG-derived or qEEG-derived outcomes related to baseline cerebral status, intradialytic change, or postdialysis recovery. Second, cognitive studies were included as complementary clinical evidence when they examined cognitive performance or neurological outcomes in maintenance hemodialysis populations and helped contextualize the clinical relevance of cerebral dysfunction. Third, imaging and hemodynamic studies were included as complementary physiological evidence when they reported CBF, cerebral oxygenation, diffusion imaging, perfusion imaging, IDH, or related cerebral physiological measures during or around hemodialysis. Fourth, systematic reviews, meta-analyses, narrative reviews, guidelines, and conceptual papers were retained only as contextual references and were not treated as primary evidence sources for qEEG findings. Fifth, engineering and methodological papers were included when they addressed EEG hardware, electrode systems, signal processing methods, artifact rejection, multimodal synchronization, wearable monitoring, edge computing, or digital biomarker validation relevant to future implementation.

Sources were excluded if they focused exclusively on peritoneal dialysis populations, involved pediatric populations, did not address EEG, qEEG, cognitive function, cerebral imaging, hemodynamic measures, or monitoring-relevant implementation issues, or consisted solely of theoretical discussion without empirical, methodological, or implementation-relevant content. Conference abstracts, editorials, commentaries, letters, and sources lacking sufficient methodological detail were excluded from the primary qualitative synthesis. Review articles and selected methodological sources were retained only for contextual interpretation or technical framework development when relevant.

### Selection of Sources of Evidence

Records identified from all databases were imported into a reference management system and screened for duplicates. After duplicate removal, titles and abstracts were screened using the predefined eligibility criteria. Potentially relevant records were then assessed in full text.

One reviewer performed the primary title, abstract, and full-text screening. A second reviewer checked the screening decisions and eligibility classifications against the predefined inclusion and exclusion criteria. Screening was therefore conducted using a reviewer-checking approach rather than fully independent duplicate screening. Disagreements or uncertainties regarding eligibility were resolved through discussion and consensus between the reviewers. When uncertainty remained, the full text was rechecked to determine whether the source contributed direct qEEG evidence, complementary cognitive or physiological evidence, contextual review evidence, or engineering-relevant implementation evidence.

A total of 916 records were initially identified. After the removal of 362 (39.5%) duplicate records, 554 (60.5%) records underwent title and abstract screening. Seventy full-text articles were assessed for eligibility, of which 8 (11.4%) articles were excluded because of insufficient EEG or qEEG data, inclusion of nonhemodialysis populations, or inadequate methodological reporting. Ultimately, 62 (88.6%) studies were included in the qualitative synthesis.

### Data Charting Process

A standardized data charting form was developed for this review to ensure consistent extraction across heterogeneous source types. The form was pilot tested on an initial subset of eligible studies and refined to improve consistency of data capture across clinical, neurophysiological, imaging, hemodynamic, and engineering-oriented publications.

For each included source, the charting form captured bibliographic information, study design, sample characteristics, dialysis-related variables, hemodialysis cycle phase, EEG or qEEG acquisition methods, signal processing procedures, reported qEEG features, cognitive or cerebral physiological outcomes, principal findings, and implementation-relevant technical information. For engineering and methodological sources, extracted items included EEG hardware configuration, electrode systems, artifact rejection methods, preprocessing pipelines, synchronization approaches, computational architecture, and validation framework relevance.

One reviewer performed the initial data charting, and a second reviewer checked the extracted information against the source articles. Discrepancies were resolved by discussion and consensus. When necessary, extracted items were revised after rechecking the full-text article to ensure that each source was assigned to the appropriate evidence role and hemodialysis cycle phase.

### Methodological Characteristics and Evidence Appraisal

As this review aimed to map heterogeneous evidence rather than evaluate intervention effectiveness or generate pooled effect estimates, a formal risk-of-bias tool was not applied. This approach is consistent with commonly accepted scoping review methodology [29-32]. Nevertheless, methodological characteristics were descriptively appraised across the included studies to support interpretation of evidence consistency, reproducibility, and translational relevance.

The appraisal focused on 6 domains: study design, sample size, EEG acquisition quality, signal processing quality, missing data reporting, and clinical heterogeneity. These domains were selected because they directly influence the reliability, reproducibility, and translational applicability of qEEG findings [18,19,22-28,33-35]. The appraisal was used for descriptive interpretation and evidence mapping, not as an exclusion criterion or a formal evidence-weighting procedure. Results of this descriptive appraisal are summarized in Table 1.

**Table 1.** Methodological domains used for descriptive evidence appraisal.

Domain	Assessment focus
Study design	Prospective, retrospective, cross-sectional
Sample size	Small (<30), moderate (30-100), large (>100)
EEG^a^ acquisition quality	Channel count, sampling frequency, reporting completeness
Signal processing quality	Filtering, artifact rejection, reproducibility
Missing data reporting	Reported, partially reported, not reported
Clinical heterogeneity	Dialysis duration, comorbidities, population variability

a EEG: electroencephalography.

### Synthesis of Results and Selection of Representative Studies

Results were synthesized narratively and organized using an evidence mapping approach. The 62 included studies were first grouped according to the 3 temporal domains of the hemodialysis cycle: predialysis baseline, intradialytic exposure, and postdialysis recovery. Within each temporal domain, findings were further mapped according to evidence role and analytical focus, including direct EEG or qEEG findings, cognitive outcomes, cerebral imaging or hemodynamic findings, and engineering- or implementation-related considerations.

Representative studies were selected for tables and narrative discussion to illustrate the main evidence domains identified in the review. Selection was based on direct relevance to the hemodialysis cycle phase, contribution to qEEG or neurophysiological interpretation, relevance to cerebral perfusion or hemodynamic mechanisms, methodological clarity, sample size or study design when applicable, and importance for the proposed translational engineering framework. Direct qEEG studies were prioritized when discussing electrophysiological findings. Cognitive, imaging, hemodynamic, review, and engineering papers were used to provide complementary physiological context or implementation guidance and were not presented as equivalent substitutes for direct qEEG evidence.

As several included studies reported overlapping findings or contributed to similar thematic domains, the representative studies presented in Table 2 and cited in the narrative discussion do not constitute an exhaustive list of all 62 included studies. Instead, they were selected to demonstrate the major clinical, physiological, neurophysiological, and engineering themes relevant to future qEEG-based cerebral monitoring during hemodialysis.

**Table 2.** Representative studies across the hemodialysis cycle and reported neurophysiological observations^a^.

Study	Hemodialysis cycle phase	Design and sample	Physiological targets	Principal findings	Reported neurophysiological observation
Kurella Tamura et al (2017) [1]	Predialysis baseline	Prospective cohort; N=314	Cognitive function	Cognitive impairment was prevalent among maintenance hemodialysis patients and associated with adverse outcomes.	Baseline cognitive dysfunction was reported among maintenance hemodialysis populations.
Dasgupta et al (2018) [6]	Intradialytic window	Clinical study; N=82	Cognitive performance (MoCA^b^)	Significant decline in cognitive scores occurred during a single hemodialysis session.	Transient cognitive deterioration was reported during dialysis treatment.
Findlay et al (2019) [16]	Intradialytic window	Prospective cohort	Cerebral perfusion and cognition	Hemodialysis-associated cerebral hypoperfusion was associated with cognitive decline and cerebrovascular injury markers.	Reductions in cerebral perfusion were observed during dialysis treatment.
Sars et al (2020) [9]	Intradialytic window	Narrative review	IDH^c^	IDH was associated with increased risk of systemic and cerebral hypoperfusion.	Cerebral hypoperfusion was reported during episodes of IDH.
Sprick et al (2020) [36]	Intradialytic window	Physiological review	Cerebral blood flow regulation	Hemodialysis-related reductions in cerebral blood flow and oxygenation were reported.	Altered cerebral autoregulation and oxygenation were described during hemodialysis.
Anazodo et al (2023) [37]	Intradialytic window	MRI^d^ or MRS^e^ study	Diffusion metrics and cerebral metabolism	Acute diffusion and metabolic abnormalities developed during hemodialysis treatment.	Imaging-derived cerebral physiological alterations were observed during dialysis.
Anazodo et al (2023) [37]	Postdialysis recovery	MRI or MRS follow-up analysis	Diffusion recovery patterns	Incomplete reversal of diffusion abnormalities was observed following hemodialysis.	Persistent physiological alterations were reported after dialysis completion.
McIntyre (2024) [38]	Postdialysis recovery or cumulative burden	Review	Recurrent circulatory stress	Repeated dialysis-associated circulatory stress was discussed in relation to cumulative multiorgan injury.	Repeated dialysis-associated circulatory stress and multiorgan physiological effects were discussed.

a As direct qEEG studies in hemodialysis remain limited, this table includes representative EEG, cognitive, neuroimaging, hemodynamic, and physiological investigations that provide complementary evidence regarding cerebral alterations across the hemodialysis cycle. Studies were selected to represent major physiological domains identified in the literature and do not constitute an exhaustive summary of all included studies.

b MoCA: Montreal Cognitive Assessment.

c IDH: intradialytic hypotension

d MRI: magnetic resonance imaging.

e MRS: magnetic resonance spectroscopy.

### Technical and Translational Framework

In addition to the clinical and neurophysiological evidence synthesis, this review incorporated technical factors relevant to the future implementation of EEG-based cerebral monitoring systems in hemodialysis settings. Particular attention was given to variability in EEG hardware, wet and dry electrode technologies, signal processing pipelines, artifact rejection methods, synchronization of multimodal physiological data streams, edge computing requirements, and digital biomarker validation frameworks [18-28,33-35].

Technical domains were extracted and synthesized to clarify implementation requirements rather than to validate a specific device, algorithm, or clinical monitoring system. The translational framework proposed in this review should therefore be regarded as a conceptual implementation model derived from the available literature. It should not be interpreted as a clinically validated monitoring architecture. This distinction is important because current evidence remains heterogeneous, and substantial prospective validation is required before routine clinical deployment can be recommended [18,19,33-35]. These technical domains and their representative implementation examples are summarized in Table 3.

**Table 3.** Technical domains extracted for translational synthesis.

Component	Examples
Hardware	Clinical EEG^a^ systems and wearable EEG platforms
Electrode systems	Wet electrodes and dry electrodes
Signal processing	Filtering, spectral analysis, and feature extraction
Artifact rejection	Independent component analysis, adaptive filtering, and wavelet denoising
Synchronization	Time-stamped EEG, hemodynamic, cerebral perfusion, and dialysis-related physiological data
Edge computing	Local artifact reduction, feature extraction, and near–real-time processing
Validation	Technical verification, analytical validation, and clinical validation

a EEG: electroencephalography.

## Results

### Study Selection

Records identified from all databases were imported into a reference management system and screened for duplicates. After duplicate removal, titles and abstracts were screened using the predefined eligibility criteria. Potentially relevant records were subsequently assessed in full text.

A total of 916 records were initially identified. After removal of 362 duplicate records, 554 records underwent title and abstract screening. Seventy full-text articles were assessed for eligibility, of which 8 were excluded because of insufficient EEG or qEEG data, inclusion of nonhemodialysis populations, or inadequate methodological reporting. Ultimately, 62 studies were included in the qualitative synthesis.

The included studies informed the evidence mapping, thematic synthesis, and development of the proposed translational framework. As multiple studies reported overlapping findings or contributed to similar evidence domains, representative publications were selectively cited in the narrative discussion to support specific methodological, technical, physiological, and clinical interpretations. Consequently, the representative studies presented in tables and cited in the narrative discussion do not constitute an exhaustive list of all 62 included studies.

**Figure 2.**
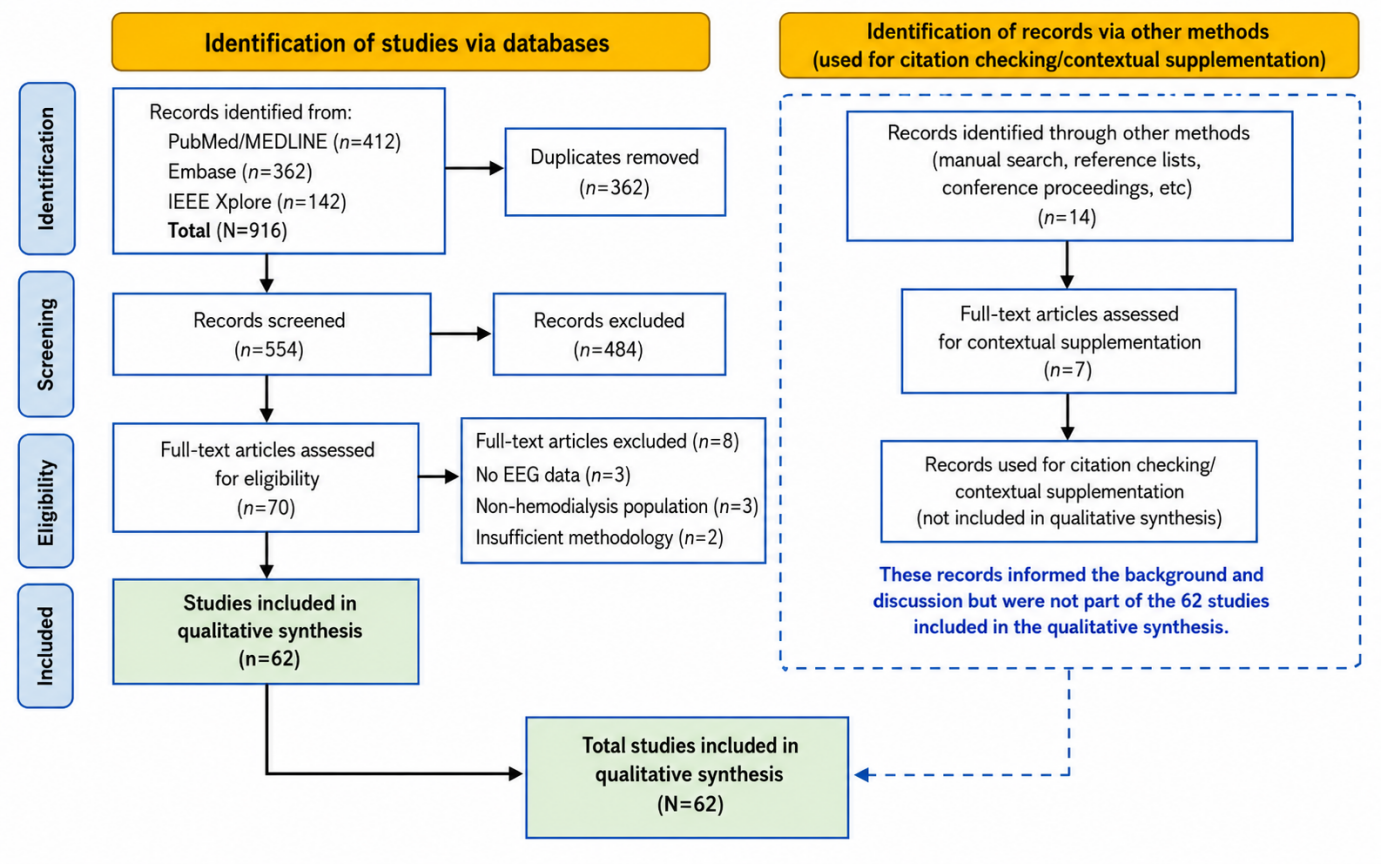
PRISMA (Preferred Reporting Items for Systematic Reviews and Meta-Analyses)–based study selection flow diagram for the scoping review of quantitative electroencephalography (EEG) in the hemodialysis cycle.

The study selection process and reasons for exclusion are summarized in Figure 2.

### Evidence Across the Hemodialysis Cycle

The included studies reported neurophysiological findings across the 3 temporal phases of the hemodialysis cycle: predialysis baseline, intradialytic exposure, and postdialysis recovery. Reported outcomes included cognitive assessments, electroencephalographic measures, cerebral perfusion parameters, neuroimaging findings, and physiological monitoring data.

Table 2 summarizes representative studies across these phases and presents study design, physiological targets, principal findings, and reported neurophysiological observations. Studies addressing predialysis baseline, intradialytic exposure, and postdialysis recovery were identified across the included literature.

### Predialysis Baseline Findings

Studies evaluating patients before dialysis treatment reported persistent neurophysiological alterations in individuals with end-stage kidney disease. Reported findings included cognitive impairment, generalized EEG slowing, altered spectral characteristics, and changes in network-related measures [1-4].

Electrophysiological studies reported patterns of spectral slowing across multiple cohorts. Early EEG investigations described generalized background slowing, whereas more recent qEEG studies have reported increased delta and theta activity together with reduced posterior alpha dominance [11-16]. Although these spectral features are reproducible across studies, their magnitude and spatial distribution differ among individuals.

Additional analyses, including complexity metrics and microstate-based approaches, reported differences in network-related measures and temporal EEG characteristics. These alterations were reported particularly among patients with metabolic burden and malnutrition-inflammation complex syndrome [13,14]. Differences in acquisition protocols, channel configurations, preprocessing pipelines, and analytical methodologies were reported across studies.

Variations in spectral characteristics, network-related measures, and complexity metrics were reported among patients undergoing maintenance hemodialysis [13,14].

### Intradialytic Findings

Studies conducted during hemodialysis treatment reported dynamic physiological and neurophysiological changes occurring throughout the dialysis session. Imaging studies using positron emission tomography, near-infrared spectroscopy, transcranial Doppler ultrasound, and magnetic resonance imaging have consistently reported reductions in CBF during ultrafiltration, typically in the range of 10% to 15%, although substantial interindividual variability exists [5-8]. More recent investigations have reported concurrent hemodynamic alterations, transient cerebral hypoperfusion, and imaging-derived abnormalities during dialysis treatment [37,39-41].

Reductions in cerebral perfusion have also been reported concurrently with transient cognitive decline and imaging-derived markers of white matter vulnerability [5,6]. Variability in physiological responses was reported across individuals and treatment sessions.

qEEG findings reported during this phase included changes in spectral indices, slow wave activity, and alpha-delta ratio (ADR). Spectral indices, particularly the ADR, were reported to decrease during periods of hemodynamic instability. Several studies reported changes in spectral indices during episodes of hemodynamic instability and IDH, although reported associations differed across studies [9,15,16].

Some studies reported gradual spectral changes during dialysis treatment, whereas others described more abrupt alterations. Associations between hemodynamic variables and EEG-derived features were reported in studies incorporating synchronized physiological measurements.

Methodological differences have also been reported across studies, including variability in EEG acquisition protocols, artifact rejection procedures, electrode systems, preprocessing workflows, and synchronization methods [20-28]. Multimodal investigations combining qEEG with cerebral perfusion imaging and hemodynamic monitoring reported concurrent changes in neurophysiological and hemodynamic variables during dialysis treatment [39-41].

### Postdialysis Findings

Studies evaluating patients after completion of hemodialysis reported variable neurophysiological findings during the postdialysis period. Reported observations included changes in physiological measures as well as persistent alterations in some individuals.

Imaging studies have reported postdialysis changes in cerebral physiological parameters following completion of treatment. Incomplete reversal of acute diffusion abnormalities and other markers of cerebral stress has also been described in some patients, with physiological alterations reported beyond the treatment session [40,41].

Available qEEG studies reported recovery-related findings following dialysis treatment. Some studies described posttreatment changes in spectral measures, whereas others reported persistent alterations in slow wave activity and network-related measures.

## Discussion

### Principal Findings

The findings synthesized in this review indicate that neurophysiological alterations associated with hemodialysis have been reported across the predialysis, intradialytic, and postdialysis phases. Building upon these observations, the following discussion examines the informatics functions of qEEG feature families and their potential roles within future cerebral monitoring frameworks.

### qEEG Feature Families and Task-Oriented Informatics Mapping

The studies identified in this review used a wide range of qEEG features to characterize neurological alterations associated with hemodialysis. Whereas the Results section summarized reported neurophysiological findings across the dialysis cycle, the present discussion focuses on the signal processing and informatics functions of qEEG features and their potential roles within different stages of hemodialysis.

For practical interpretation, qEEG features can be broadly organized into 4 major families: spectral power features, connectivity metrics, complexity measures, and temporal dynamic descriptors. Each feature family captures different aspects of brain activity and therefore provides distinct information about cerebral physiology. Rather than serving as interchangeable representations of neural function, these features address different monitoring objectives and may exhibit varying sensitivity to dialysis-related physiological disturbances.

From an informatics perspective, the selection of qEEG features should be guided by the intended analytical task. Spectral power measures are commonly used to characterize cerebral states and detect frequency-specific alterations associated with cognitive dysfunction or metabolic disturbances. Connectivity metrics provide information regarding large-scale functional network organization, whereas complexity measures quantify signal irregularity and information content. Temporal dynamic descriptors, including EEG microstates and state transition patterns, may further capture transient changes and evolving neurological trajectories during treatment.

Feature selection must also be considered within the operational environment of hemodialysis. Motion artifacts, electrical interference from dialysis equipment, electrode stability, synchronization with physiological measurements, and real-time processing requirements can all influence feature reliability and clinical interpretability. Accordingly, evaluation of qEEG biomarkers should extend beyond physiological relevance to include signal quality, implementation feasibility, and validation considerations.

The following sections review these feature families individually and discuss their relationships with monitoring objectives, implementation requirements, and translational informatics tasks. A task-oriented mapping framework is subsequently proposed to illustrate how different qEEG feature families may support cerebral monitoring, event detection, risk stratification, patient phenotyping, and longitudinal assessment throughout the hemodialysis process.

### Spectral Power Features: State Tracking and Event Sensitivity

Spectral power analysis represents the most widely applied qEEG feature family in studies of neurological monitoring during hemodialysis. These features quantify the distribution of EEG signal energy across conventional frequency bands and provide a relatively simple yet physiologically interpretable representation of cerebral activity. Commonly reported metrics include absolute and relative spectral power within the delta, theta, alpha, beta, and gamma bands, as well as derived indices such as the theta-alpha ratio and the ADR.

Across the reviewed studies, spectral features were consistently associated with dialysis-related alterations in cerebral function. Increases in slow wave activity, particularly within the delta and theta bands, together with reductions in alpha activity or ADR, were frequently observed during periods of hemodynamic instability, cerebral hypoperfusion, or acute physiological stress associated with hemodialysis [13,15,16]. These findings are broadly consistent with established EEG manifestations of metabolic encephalopathy and cerebral dysfunction, in which slowing of background activity reflects impaired neuronal regulation and reduced cortical efficiency [11,12].

From an informatics perspective, spectral power measures are particularly suited for state tracking applications. As spectral estimates can be computed continuously over successive analysis windows, they enable longitudinal assessment of cerebral status throughout the dialysis session. Progressive shifts toward slower frequency components may indicate evolving physiological stress, whereas stabilization or recovery of spectral profiles may reflect the restoration of cerebral homeostasis following treatment-related perturbations.

Several studies further suggested that spectral alterations may occur in temporal association with clinically important events, including IDH, dialysis disequilibrium syndrome, and episodes of cerebral hypoperfusion [6,7,16]. Although current evidence remains insufficient to establish robust predictive biomarkers, these observations support the potential role of spectral features in event-sensitive monitoring frameworks. In such applications, EEG-derived spectral changes would be interpreted together with synchronized physiological variables, including blood pressure, heart rate, ultrafiltration volume, and cerebral perfusion indicators, rather than as stand-alone diagnostic markers.

An additional advantage of spectral power features is their compatibility with real-time and resource-constrained monitoring systems. Fast Fourier transform–based spectral estimation requires relatively modest computational resources and can be implemented in wearable EEG platforms, bedside monitoring systems, or edge computing architectures designed for continuous neurophysiological surveillance [27,42,43]. This characteristic makes spectral features attractive candidates for future translational monitoring systems in dialysis environments.

Nevertheless, interpretation of spectral measures requires careful consideration of signal quality and artifact contamination. Motion-related artifacts, muscle activity, electrode impedance fluctuations, and electrical interference generated by dialysis equipment may influence spectral estimates and potentially mimic physiological changes. Appropriate preprocessing, artifact rejection, and synchronization procedures are therefore essential to ensure the analytical validity and clinical interpretability of spectral biomarkers.

### Connectivity Metrics: Network-Level Characterization

Connectivity metrics describe interactions among distributed brain regions and provide information that extends beyond localized oscillatory activity. Unlike spectral power features, which quantify signal energy within individual frequency bands, connectivity measures evaluate the degree of functional coupling between spatially separated neural populations. Commonly reported metrics include coherence, phase-locking value, synchronization indices, mutual information, and other measures of functional integration.

In the reviewed literature, connectivity-related abnormalities were primarily associated with cognitive dysfunction and chronic neurological burden in hemodialysis populations. Altered coherence patterns and disrupted network organization have been reported in patients exhibiting cognitive impairment, malnutrition-inflammation complex syndrome, and other conditions linked to long-term cerebral vulnerability [13,14]. In particular, reductions in theta-band coherence have been interpreted as evidence of impaired large-scale neural communication, although the magnitude and spatial distribution of these findings vary across studies.

Compared with spectral power measures, connectivity features may capture more persistent aspects of cerebral dysfunction. Several investigations suggested that network-level alterations can remain detectable even when systemic physiological variables appear stable following dialysis treatment [5,6]. This observation raises the possibility that connectivity metrics reflect cumulative neurological stress and chronic network reorganization rather than only transient intradialytic physiological fluctuations.

Several reviews have further suggested that repeated dialysis-related circulatory stress may contribute to cerebral vulnerability and long-term neurological burden in hemodialysis populations [37,38]. Within this context, connectivity-related abnormalities may provide a network-level perspective on chronic neurophysiological alterations associated with repeated hemodynamic and metabolic stressors. However, the available evidence remains heterogeneous, and the mechanistic relationships between dialysis-related physiological stress and connectivity changes have not yet been fully established.

From a task-oriented informatics perspective, connectivity features are most relevant for cognitive phenotyping and longitudinal trajectory assessment. As functional network organization may evolve gradually over repeated dialysis sessions, connectivity measures may provide complementary information regarding disease progression, treatment response, and interindividual heterogeneity. These characteristics make connectivity analysis potentially valuable for identifying patient subgroups with differing neurological risk profiles.

Despite these advantages, connectivity analysis imposes substantially greater technical requirements than conventional spectral monitoring. Reliable estimation requires stable electrode placement, adequate spatial sampling, consistent referencing strategies, and rigorous artifact suppression. Motion artifacts, electrode displacement, muscle activity, and electrical interference can artificially inflate or suppress connectivity estimates, leading to spurious network interpretations. Furthermore, connectivity measures often require longer analysis windows and greater computational resources than standard spectral calculations, which may limit their applicability in real-time monitoring environments.

Consequently, although connectivity metrics offer unique insights into large-scale brain network organization, their translation into routine dialysis monitoring will require standardized acquisition protocols, robust signal processing pipelines, and validation across diverse patient populations. Future implementation efforts should also evaluate whether simplified network biomarkers can retain clinical utility while remaining compatible with wearable EEG platforms and continuous monitoring systems.

### Complexity Measures: Signal Organization and State Compression

Complexity-based qEEG features quantify the degree of irregularity, unpredictability, and information richness within neural signals. Unlike spectral power measures, which characterize the distribution of signal energy across frequency bands, complexity metrics evaluate how neural activity is organized over time and therefore provide complementary information regarding brain function. Commonly reported measures include Shannon entropy, sample entropy, approximate entropy, and Lempel-Ziv complexity, each reflecting different aspects of signal variability and informational structure.

In neurological and metabolic disorders, reduced EEG complexity has frequently been associated with impaired cerebral function, diminished adaptive capacity, and the loss of dynamic neural organization [11,12]. Within the hemodialysis setting, complexity reductions may occur in response to cerebral hypoperfusion, metabolic instability, or dialysis-related physiological stress [16]. These alterations are thought to reflect a transition toward more stereotyped and less flexible patterns of cortical activity. Consequently, complexity measures may reveal aspects of neurophysiological dysfunction that are not fully captured by conventional frequency-domain analyses.

From a task-oriented informatics perspective, complexity metrics are particularly attractive because they condense high-dimensional EEG signals into compact quantitative descriptors. This characteristic supports continuous monitoring and facilitates longitudinal comparison across treatment sessions. Rather than focusing on specific oscillatory bands, complexity measures provide a global index of signal organization, making them potentially useful for tracking overall neurological status, identifying gradual deterioration, and characterizing patient-specific trajectories over time.

Complexity features may also contribute to multimodal biomarker development when combined with spectral, connectivity, and physiological measurements. As complexity metrics summarize information content at the signal level, they can complement frequency-based and network-based analyses, potentially improving the robustness of composite monitoring frameworks. Such integration may be especially valuable in hemodialysis environments where multiple physiological systems are simultaneously affected by fluid shifts, hemodynamic stress, and metabolic fluctuations.

From an implementation perspective, entropy-based and compression-based calculations generally require modest computational resources and can be incorporated into real-time signal processing pipelines. Their compact output representation is particularly compatible with edge computing architectures and wearable monitoring platforms, where computational efficiency and bandwidth constraints are important considerations. However, complexity estimates may be sensitive to signal length, preprocessing strategies, and noise contamination. Further validation is therefore required to establish reproducible acquisition protocols, clinically meaningful thresholds, and analytical performance benchmarks before complexity metrics can be considered reliable digital biomarkers for routine dialysis monitoring.

### Temporal Dynamics: Microstates and Trajectory Analysis

Temporal dynamic features characterize how brain activity evolves over time rather than describing static signal properties measured within isolated analysis windows. Whereas spectral, connectivity, and complexity measures summarize specific aspects of neural activity at a given moment, temporal features focus on transitions, persistence, and recovery patterns across time. This category includes EEG microstates, state transition metrics, temporal variability measures, and trajectory-based descriptors that capture the dynamic organization of cerebral activity.

Among these approaches, EEG microstate analysis has received increasing attention as a framework for investigating large-scale brain dynamics. Microstates represent short-lasting, quasi-stable spatial configurations of scalp electrical activity that typically persist for tens of milliseconds before transitioning to another state. Quantitative descriptors such as microstate duration, occurrence frequency, coverage, and transition probability provide information regarding the temporal organization of neural processing.

Alterations in microstate dynamics have been reported in association with cognitive impairment, metabolic disturbances, and inflammatory conditions, including malnutrition-inflammation complex syndrome and uremic burden in hemodialysis populations [13,14]. Although the specific microstate patterns reported across studies are not entirely consistent, these findings suggest that the temporal organization of brain activity may be sensitive to physiological perturbations that are not fully reflected by conventional spectral measures. Temporal features therefore offer a complementary perspective on cerebral dysfunction by emphasizing dynamic behavior rather than static signal characteristics.

From a task-oriented informatics perspective, temporal dynamic features are particularly relevant for trajectory analysis and recovery assessment. As hemodialysis involves repeated physiological stress and recovery cycles, temporal metrics may help characterize how cerebral activity changes during treatment, stabilizes following intervention, and varies across consecutive dialysis sessions. Such analyses may provide insight into treatment-related resilience, cumulative neurological burden, and interindividual variability in recovery patterns.

Unlike severe neurological conditions in which burst suppression or profound discontinuity may occur, temporal changes observed in stable outpatient hemodialysis populations are generally more subtle and are likely to manifest as alterations in state transition behavior, temporal variability, or microstate organization. Consequently, longitudinal monitoring strategies may be more informative than isolated single-session measurements when evaluating the clinical relevance of temporal biomarkers.

Implementation of temporal dynamic analysis also introduces additional methodological considerations. Reliable estimation requires continuous recordings of sufficient duration, precise temporal synchronization, and consistent acquisition protocols across sessions. Variations in recording length, preprocessing pipelines, and artifact handling procedures may substantially influence temporal metrics and complicate comparisons between studies. Future validation efforts should therefore focus on standardizing temporal analysis frameworks and determining whether trajectory-based biomarkers provide incremental value beyond conventional spectral and connectivity measures.

As temporal features explicitly model changes over time, they are conceptually well aligned with continuous monitoring systems and longitudinal digital biomarker frameworks. As wearable EEG technologies and edge computing platforms continue to evolve, temporal dynamic analysis may become increasingly important for assessing cerebral adaptation, recovery, and neurological stability throughout the hemodialysis cycle.

### Mapping Feature Families to Informatics Tasks

The preceding sections demonstrate that individual qEEG feature families provide distinct yet complementary representations of cerebral function. Rather than serving as interchangeable biomarkers, spectral, connectivity, complexity, and temporal dynamic features address different analytical objectives and operate at different levels of neurophysiological abstraction. Consequently, selection of qEEG features should be guided by the intended monitoring task, available computational resources, signal quality requirements, and clinical context.

As summarized in Table 4, spectral power features are most closely aligned with cerebral state monitoring and event-sensitive surveillance. As spectral measures can be computed efficiently and updated continuously, they are well suited for applications requiring near–real-time assessment of intradialytic physiological changes. In contrast, connectivity metrics emphasize network-level organization and are more commonly associated with cognitive phenotyping, patient stratification, and longitudinal evaluation of functional brain integrity. Although connectivity measures may provide richer information regarding large-scale neural interactions, they typically require more stringent acquisition and preprocessing conditions.

**Table 4.** Mapping of quantitative electroencephalography feature families to informatics tasks and deployment considerations.

Feature family	Primary informatics task	Representative monitoring objective	Dominant hemodialysis cycle phase	Deployment considerations	Validation maturity
Spectral power	State tracking and event-sensitive monitoring	Detection of cerebral stress and IDH^a^-associated alterations	Intradialytic	Low computational demand; suitable for real-time monitoring and edge deployment	Moderate
Connectivity	Cognitive phenotyping and network characterization	Assessment of chronic neurological burden and cognitive dysfunction	Pre- and postdialysis	Requires stable electrode placement and robust artifact suppression	Exploratory
Complexity	Neurophysiological state indexing	Continuous assessment of signal organization and cerebral stability	All phases	Computationally efficient; suitable for wearable and continuous monitoring systems	Exploratory
Temporal dynamics	Trajectory analysis and recovery modeling	Characterization of recovery patterns and intersession variability	Postdialysis and longitudinal follow-up	Requires repeated recordings and consistent acquisition protocols	Early stage

a IDH: intradialytic hypotension.

Complexity measures occupy an intermediate position within the monitoring framework. By condensing EEG activity into compact descriptors of signal organization and information content, complexity metrics facilitate continuous tracking of neurological status while remaining computationally efficient. These properties make them attractive candidates for integration into edge computing and wearable monitoring platforms. Temporal dynamic features extend this framework by explicitly characterizing how cerebral activity evolves over time. Measures derived from EEG microstates, state transition patterns, and temporal variability may therefore be particularly informative for recovery assessment, trajectory analysis, and repeated session monitoring.

Importantly, no single feature family appears sufficient to capture the full spectrum of neurological responses associated with hemodialysis. Spectral slowing, network disruption, complexity reduction, and temporal reorganization may reflect related but nonidentical physiological processes. A task-oriented framework therefore favors complementary feature integration rather than reliance on individual biomarkers. Such an approach is consistent with contemporary digital biomarker development strategies, in which multiple signal representations are combined to improve analytical robustness and clinical interpretability [17-19].

From a translational informatics perspective, the mapping presented in Table 4 should be viewed as a proposed implementation framework rather than as a validated clinical monitoring system. The current evidence base remains heterogeneous with respect to study design, EEG acquisition protocols, signal processing methods, and patient populations. Accordingly, the proposed mapping is intended to guide future system development, hypothesis generation, and validation studies aimed at establishing reliable qEEG-based monitoring approaches for hemodialysis-related neurological assessment.

Future monitoring architectures may incorporate multiple feature families within hierarchical analytical pipelines. For example, computationally efficient spectral and complexity measures could support continuous bedside surveillance, whereas connectivity and temporal dynamic analyses could be performed periodically to provide a deeper characterization of network integrity and recovery trajectories. Such tiered approaches may help balance computational efficiency, interpretability, and clinical utility in real-world dialysis environments.

### Hardware, Signal Processing, and Validation Considerations

Although qEEG has demonstrated potential for cerebral monitoring during hemodialysis, successful translation into routine clinical practice requires consideration of hardware design, signal processing methodology, and validation requirements in addition to biomarker performance. These implementation factors are particularly important because the hemodialysis environment presents unique operational challenges, including prolonged monitoring periods, patient movement, electrical interference from dialysis equipment, and the need for synchronization with physiological measurements.

From a hardware perspective, most published studies have used conventional wet-electrode EEG systems because of their established signal quality and widespread clinical use. However, wet electrodes require skin preparation, conductive gel application, and periodic maintenance, which may limit their practicality for repeated monitoring during routine dialysis sessions. Emerging dry-electrode and wearable EEG systems offer potential advantages in terms of ease of deployment, patient comfort, and scalability for longitudinal monitoring, although signal quality and motion sensitivity remain important considerations [20,21,36,44]. Future implementation frameworks may therefore require balancing signal fidelity against operational feasibility depending on the intended monitoring application.

Signal processing pipelines represent another critical component of qEEG-based monitoring systems. Although processing strategies vary across studies, a typical workflow includes signal acquisition, filtering, artifact suppression, feature extraction, and analytical interpretation. Preprocessing procedures are particularly important because hemodialysis environments may introduce artifacts arising from patient movement, muscle activity, electrode displacement, and electrical noise generated by clinical equipment. Common approaches include independent component analysis, adaptive filtering, wavelet-based denoising, and automated artifact rejection techniques [22-25,43]. Variability in preprocessing strategies may substantially influence downstream feature estimates and complicate comparisons across studies.

Synchronization between EEG recordings and physiological measurements is also essential for meaningful interpretation of cerebral responses during dialysis. Many clinically relevant events, including IDH, rapid ultrafiltration, blood pressure fluctuations, and cerebral perfusion changes, evolve dynamically throughout treatment. Accurate temporal alignment of EEG data with hemodynamic and dialysis-related parameters therefore enables more precise investigation of causal relationships and event-associated neurophysiological responses. Future monitoring systems may benefit from integrated acquisition architectures that combine neurophysiological, hemodynamic, and treatment-related data streams within a unified analytical framework.

Variability in reported EEG-hemodynamic relationships may partly reflect differences in acquisition quality, synchronization procedures, preprocessing workflows, and study design. These factors may contribute to inconsistencies in the strength, direction, and reproducibility of reported associations across studies and further emphasize the need for standardized methodological frameworks.

Recent advances in wearable sensing and edge computing further expand opportunities for continuous cerebral monitoring. Resource-efficient qEEG features such as spectral power and complexity measures can be computed locally using embedded processors, thereby reducing data transmission requirements and supporting near–real-time analysis [26-28,42]. More computationally intensive analyses, including connectivity estimation and temporal dynamic modeling, may be performed periodically or through hybrid cloud-assisted architectures. Such hierarchical processing strategies may improve scalability while maintaining analytical flexibility in clinical environments.

Equally important is the need for rigorous validation before qEEG-derived biomarkers can be considered clinically actionable. Contemporary digital biomarker frameworks emphasize 3 complementary stages of evaluation: verification, analytical validation, and clinical validation [17-19,33-35]. Verification assesses whether hardware and software systems function as intended, analytical validation evaluates the accuracy and reliability of derived measurements, and clinical validation determines whether the resulting biomarkers are meaningfully associated with relevant clinical outcomes. Although several qEEG features have shown promising associations with cognitive dysfunction, cerebral hypoperfusion, and dialysis-related physiological stress, the available evidence remains heterogeneous with respect to study design, sample size, acquisition protocols, and outcome definitions.

Accordingly, current evidence should be viewed as supportive of future development rather than definitive clinical implementation. Standardized acquisition procedures, transparent signal processing workflows, reproducible validation protocols, and multicenter evaluation studies will be necessary before qEEG-based monitoring systems can be integrated into routine hemodialysis care. These requirements are consistent with broader recommendations for digital biomarker development and highlight the importance of engineering validation alongside clinical investigation.

### Translational Pathways Toward Clinical Implementation

The studies identified in this scoping review demonstrate growing interest in the application of qEEG for assessing cerebral responses during hemodialysis. Existing evidence suggests that qEEG may capture neurophysiological alterations associated with cerebral hypoperfusion, intradialytic stress, and cognitive dysfunction. However, the current literature remains fragmented, with substantial variability in study design, sample size, EEG acquisition protocols, feature selection strategies, and outcome definitions.

Several translational challenges remain before qEEG can be systematically evaluated as a practical monitoring approach in routine dialysis care. Most published studies have been conducted under research-specific conditions using heterogeneous hardware platforms and signal processing methodologies. In addition, longitudinal validation is limited, and synchronized acquisition of dialysis-related physiological parameters and neurophysiological signals has rarely been implemented. These limitations restrict direct comparison across studies and complicate the assessment of clinical applicability. Furthermore, the co-occurrence of cerebral hypoperfusion and imaging-derived abnormalities has been discussed as a potential indicator of dialysis-related cerebral vulnerability, although causal relationships remain incompletely established [37,39-41]. These observations highlight the need for monitoring approaches capable of capturing dynamic neurophysiological responses during dialysis treatment.

At the same time, recent advances in wearable EEG technologies, artifact reduction techniques, edge computing, and multimodal physiological monitoring have created new opportunities for continuous neurophysiological assessment in dialysis environments. Rather than representing a clinically validated deployment model, the following sections synthesize key engineering considerations identified from the current evidence base and outline potential pathways that may support future development, validation, and implementation of qEEG-based dialysis-brain monitoring systems.

**Figure 3.**
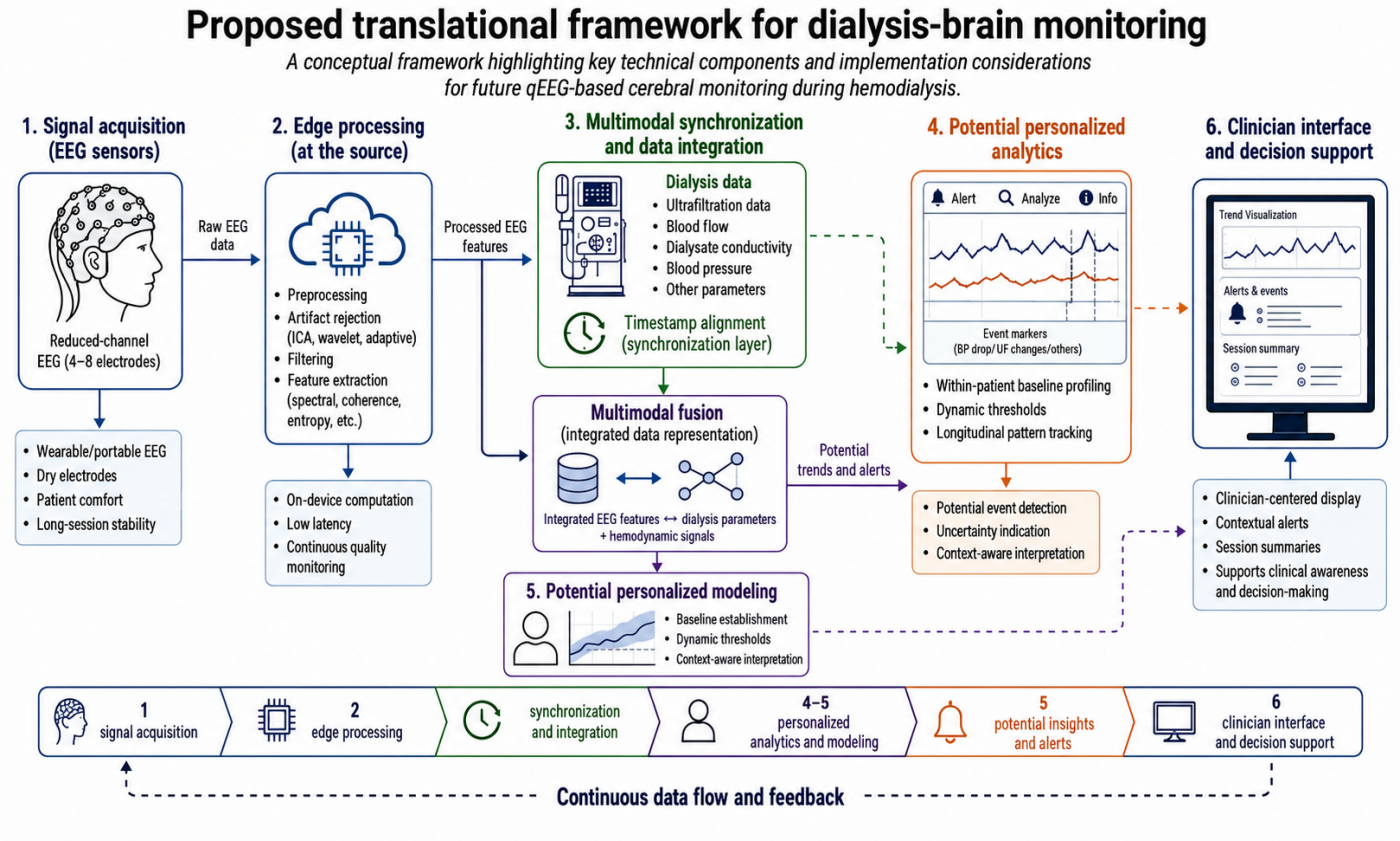
Proposed translational framework illustrating potential technical components and implementation considerations for future quantitative electroencephalography (qEEG)–based cerebral monitoring during hemodialysis. BP: blood pressure; ICA: independent component analysis; UF: ultrafiltration.

A proposed translational framework is presented in Figure 3 to illustrate the major technical components and implementation considerations that could facilitate future integration of neurophysiological monitoring into hemodialysis care.

The framework shown in Figure 3 is conceptual and intended to summarize implementation considerations identified from the literature. It should not be interpreted as a clinically validated monitoring architecture.

### Current Evidence Gaps

Although the studies identified in this review consistently suggest that hemodialysis is associated with measurable neurophysiological alterations, the current evidence base remains insufficient to support routine implementation of qEEG-based cerebral monitoring in clinical practice. Several methodological and technical limitations were observed across the included studies.

First, most investigations were conducted using relatively small cohorts and single-center designs, limiting the generalizability across diverse hemodialysis populations. Cognitive status, dialysis vintage, comorbid conditions, and treatment protocols varied substantially between studies, introducing considerable clinical heterogeneity that complicates cross-study comparisons [1-4,37,38]. In addition, many studies used cross-sectional or single-session designs, making it difficult to evaluate longitudinal changes in cerebral function or determine the stability of qEEG-derived biomarkers over time [5,6,39].

Second, substantial variability exists in EEG acquisition methodologies. Differences in electrode type, channel configuration, recording duration, sampling frequency, and artifact management procedures were frequently observed across studies. Such methodological inconsistency may contribute to variability in reported findings and hinder the direct comparison of qEEG features between cohorts [12,20,21,42]. Standardization of EEG acquisition and reporting procedures remains an important requirement for future multicenter investigations.

Third, signal processing approaches varied considerably among studies. While spectral indices were the most commonly reported biomarkers, connectivity measures, entropy metrics, and microstate analyses were applied less consistently. Differences in preprocessing pipelines, artifact rejection strategies, and feature selection methods may influence biomarker reproducibility and limit external validation [22-28,42]. Recent reporting initiatives have emphasized the importance of transparent documentation of EEG processing workflows and biomarker evaluation procedures [43].

Fourth, evidence regarding postdialysis recovery remains comparatively limited. Heterogeneity in acquisition protocols and analytical approaches limits direct comparison of recovery-related findings across studies. Furthermore, the duration, magnitude, and clinical significance of postdialysis physiological alterations remain incompletely characterized and require further longitudinal investigation [40,41]. These limitations restrict understanding of recovery trajectories and their potential relationship to cumulative neurological burden.

Another important limitation is the limited integration of neurophysiological and dialysis-related physiological data. Most studies analyzed EEG recordings independently of dialysis machine parameters, blood pressure trends, or cerebral perfusion measurements. Consequently, the temporal relationships between hemodynamic fluctuations and qEEG alterations remain incompletely characterized [5-8,39-41]. The absence of synchronized multimodal monitoring represents a significant barrier to understanding dialysis-brain interactions in real-world clinical settings.

Taken together, these evidence gaps highlight the need for larger longitudinal studies, standardized EEG acquisition and analysis protocols, multimodal data integration, and rigorous validation frameworks. Addressing these limitations will be essential before qEEG-derived biomarkers can be systematically evaluated for future clinical monitoring applications in hemodialysis care.

### Engineering Requirements for Future Systems

Translating qEEG from research studies to routine hemodialysis settings will require coordinated advances across hardware, signal processing, synchronization, and computational infrastructure. Dialysis units impose practical constraints, including multihour recordings, patient movement, electrical interference, and changing hemodynamic conditions. These factors should be considered during system design and validation.

Wearable or reduced-channel EEG configurations may reduce patient burden while maintaining sufficient signal quality. Dry-electrode and portable EEG systems can simplify setup compared with conventional wet-electrode systems, but they may introduce trade-offs in impedance stability, motion sensitivity, and signal fidelity [20,21,26,27]. The optimal configuration will depend on whether the intended application is continuous bedside surveillance, periodic assessment, or research-grade characterization.

Reliable long-duration monitoring will also require automated artifact management and signal quality assessment. Future pipelines should be able to detect movement, muscle activity, electrode displacement, and environmental noise during dialysis sessions while preserving the interpretability of derived features [22-28]. Synchronized acquisition of EEG, blood pressure, ultrafiltration rate, cerebral perfusion data, and other dialysis-related variables will be important for interpreting transient neurophysiological changes [39-41].

Computational architecture should match the monitoring objective. Edge processing may support low-latency artifact reduction and extraction of efficient spectral or complexity features, whereas connectivity and temporal trajectory analyses may require periodic offline or cloud-assisted processing [26,27]. Regardless of the architecture, future systems will require verification, analytical validation, clinical validation, reproducibility assessment, and transparent reporting before they can be evaluated for routine clinical use [17-19,33-35,43]. These requirements highlight key technical domains for future development but do not constitute a finalized deployment architecture.

### Clinical Integration and Interpretability

Clinical implementation will depend on whether qEEG-derived information can be presented in a form that is understandable and useful within routine dialysis workflows. Outputs should emphasize physiological interpretation rather than algorithmic complexity, such as trends in spectral indices, deviations from patient-specific baselines, synchronized displays of qEEG and hemodynamic variables, and event-based summaries of potential cerebral stress.

Interpretability is particularly important when advanced computational or AI-assisted methods are used. qEEG changes may be influenced by sleep state, medication use, fatigue, metabolic status, or preexisting neurological conditions, in addition to dialysis-related cerebral stress [7,28]. Monitoring systems should therefore communicate uncertainty and avoid presenting algorithmic outputs as definitive indicators of cerebral dysfunction.

Workflow compatibility is also essential. Systems requiring extensive setup, specialized interpretation, or major changes to dialysis-unit routines may be difficult to adopt even if technically feasible. Future implementation should therefore apply human-centered design principles and maintain appropriate clinical oversight [33-36,44,45].

### Ethical and Governance Considerations

Future qEEG-based monitoring in hemodialysis should be governed as an investigational physiological monitoring approach rather than as a validated decision support system. qEEG outputs may assist in identifying cerebral stress or recovery patterns, but they should not be interpreted as stand-alone diagnostic conclusions or used to replace clinical judgment. Implementation will require human oversight, transparent reporting of signal processing methods, appropriate management of uncertainty, and safeguards for neurophysiological data privacy. As continuous EEG data may contain sensitive information about cognitive and neurological status, future studies should address data security, access control, secondary use, and equitable performance across diverse dialysis populations. These safeguards are consistent with broader recommendations for digital biomarker development and trustworthy clinical AI systems [17-19,33-35].

### Future Research Priorities and Translational Outlook

The current evidence supports continued investigation of qEEG for studying dialysis-related neurophysiological responses, but it remains insufficient for routine clinical application. Future studies should prioritize standardized acquisition protocols, transparent preprocessing workflows, consistent biomarker definitions, and reproducible reporting to improve comparability across studies [18,19,42,43].

Multimodal and longitudinal designs are especially important. Synchronized acquisition of qEEG, hemodynamic variables, cerebral perfusion measures, dialysis machine parameters, and cognitive outcomes may clarify the temporal relationships between treatment-related physiological stress and cerebral responses [5-9,39-41]. As hemodialysis populations are heterogeneous, patient-specific baselines and longitudinal trajectories should also be evaluated as alternatives or complements to universal thresholds [1-4,37,38].

Advances in wearable EEG, edge computing, automated artifact management, and digital biomarker science may support future implementation [20,21,26,27]. However, technical feasibility alone is insufficient. Prospective validation, workflow integration, interpretability, governance, and outcome-based evaluation will be necessary before qEEG-based monitoring can be considered for routine hemodialysis care [17-19,33-36,44-46]. The framework proposed in this review should therefore be viewed as a conceptual guide for future validation rather than as a clinically established monitoring system.

### Conclusions

This scoping review synthesized current evidence regarding qEEG for cerebral monitoring in patients undergoing hemodialysis and examined its potential role within future translational monitoring frameworks. Across the reviewed literature, neurophysiological alterations were consistently associated with chronic kidney disease, cerebral hypoperfusion, intradialytic physiological stress, and cognitive dysfunction. However, substantial variability was observed across studies with respect to patient populations, EEG acquisition methodologies, signal processing strategies, and reported biomarkers.

The findings indicate that qEEG-derived features should be interpreted as dynamic indicators of neurophysiological responses rather than as static markers of cerebral dysfunction. Spectral slowing, alterations in alpha-related activity, and changes in network-level metrics have been reported across different stages of the hemodialysis cycle, although their temporal behavior and clinical significance remain incompletely understood. Current evidence does not support the use of universal population-based thresholds, highlighting the importance of individualized interpretation and longitudinal assessment.

From an engineering perspective, successful translation of qEEG into hemodialysis settings will require advances in wearable EEG technologies, artifact management, multimodal synchronization, edge-based processing, and validation methodologies. The technical synthesis presented in this review highlights important trade-offs involving signal acquisition, computational architecture, physiological data integration, and clinical usability. These considerations extend beyond biomarker identification and emphasize the need for robust monitoring infrastructures capable of operating within routine dialysis environments.

To facilitate future development, this review proposes a translational framework that integrates evidence gaps, engineering requirements, individualized baseline modeling, and clinician-centered implementation considerations. The framework is intended as a conceptual model derived from currently available evidence rather than as a validated clinical monitoring system. Its purpose is to guide future research efforts aimed at bridging the gap between experimental qEEG investigations and potential clinical applications.

Overall, the current evidence supports continued investigation of qEEG as a promising tool for studying dialysis-brain interactions. Future progress will depend on methodological standardization, multimodal physiological integration, prospective longitudinal validation, and human-centered implementation research. Through these efforts, qEEG-based approaches may contribute to a deeper understanding of dialysis-related cerebral dysfunction and inform the development of future neurophysiological monitoring strategies in hemodialysis care.

## Supplementary material

10.2196/94560 Multimedia Appendix 1 .Database-specific search strategies for PubMed/MEDLINE, Embase, and IEEE Xplore used in this scoping review

10.2196/94560 Checklist 1 PRISMA-ScR checklist.

## Abbreviations

ADR: alpha-delta ratio
CBF: cerebral blood flow
EEG: electroencephalography
IDH: intradialytic hypotension
PRISMA-ScR: Preferred Reporting Items for Systematic Reviews and Meta-Analyses extension for Scoping Reviews
qEEG: quantitative electroencephalography
