# Supplementary material for: Quantitative Electroencephalography in Hemodialysis: Scoping Review and Translational Framework
Source: JMIR Biomed Eng. 2026 Jul 31;11:e94560. doi: 10.2196/94560 (PMC13427059; doi:10.2196/94560)
Supplement: Multimedia Appendix 1 [file biomedeng-v11-e94560-s001.pdf]

Multimedia Appendix 1. Database-Specific Search Strategies

Title: Quantitative Electroencephalography in Hemodialysis: Scoping Review and Translational Framework

Search Period

The searches covered records published from 2005 through 2026 and were last conducted on February 28, 2026.

Information Sources

The following electronic databases were searched:

1. PubMed/MEDLINE
2. Embase
3. IEEE Xplore

Search Framework

The database searches were structured around three main concept domains:

(1) the hemodialysis population, including hemodialysis, haemodialysis, end-stage kidney disease, end-stage renal disease, ESKD, and ESRD;

(2) electroencephalography and quantitative EEG, including electroencephalography, EEG, quantitative EEG, qEEG, spectral analysis, functional connectivity, and EEG microstates; and

(3) cerebral and cognitive outcomes, including cognitive impairment, cognitive dysfunction, cerebral blood flow, cerebral perfusion, uremic encephalopathy, intradialytic hypotension, and brain injury.

Additional engineering-oriented searches were used to explore translational topics related to wearable EEG, electrodes, signal processing, artifact rejection, physiological monitoring, edge computing, and digital biomarkers.

PubMed/MEDLINE Search Strategy

(("hemodialysis"[Title/Abstract]
OR "haemodialysis"[Title/Abstract]
OR "end-stage kidney disease"[Title/Abstract]
OR "end-stage renal disease"[Title/Abstract]
OR ESKD[Title/Abstract]
OR ESRD[Title/Abstract])

AND

("electroencephalography"[Title/Abstract]
OR EEG[Title/Abstract]
OR "quantitative EEG"[Title/Abstract]
OR qEEG[Title/Abstract]
OR "spectral analysis"[Title/Abstract]
OR "functional connectivity"[Title/Abstract]
OR microstate*[Title/Abstract])

AND

("cognitive impairment"[Title/Abstract]
OR "cognitive dysfunction"[Title/Abstract]
OR "cerebral blood flow"[Title/Abstract]
OR "cerebral perfusion"[Title/Abstract]
OR "uremic encephalopathy"[Title/Abstract]
OR "intradialytic hypotension"[Title/Abstract]
OR "brain injury"[Title/Abstract]))

Publication date filter: January 1, 2005, to February 28, 2026.

Embase Search Strategy

('hemodialysis'/exp
OR 'haemodialysis'/exp
OR 'end stage kidney disease'/exp
OR 'end stage renal disease'/exp
OR 'ESKD'
OR 'ESRD')

AND

('electroencephalography'/exp
OR 'EEG'
OR 'quantitative EEG'
OR 'qEEG'
OR 'spectral analysis'
OR 'functional connectivity'
OR 'microstate')

AND

('cognitive impairment'/exp
OR 'cognitive dysfunction'/exp
OR 'cerebral blood flow'/exp
OR 'cerebral perfusion'/exp
OR 'uremic encephalopathy'/exp
OR 'intradialytic hypotension'/exp
OR 'brain injury'/exp)

Publication year limit: 2005–2026, with the final search conducted on February 28, 2026.

IEEE Xplore Search Strategy

("electroencephalography"
OR "EEG"
OR "quantitative EEG"
OR "qEEG")

AND

("hemodialysis"
OR "haemodialysis"
OR "end-stage kidney disease"
OR "renal failure")

Publication year limit: 2005–2026, with the final search conducted on February 28, 2026.

Additional engineering-focused searches were conducted to support the translational framework and included combinations of:

("wearable EEG"
OR "dry electrode"
OR "signal processing"
OR "artifact rejection"
OR "edge computing"
OR "digital biomarker")

AND

("physiological monitoring"
OR "clinical monitoring")

These additional engineering-focused searches were used to inform the translational

framework and were not included in the PRISMA study-selection counts.

Screening and Study Selection

Records retrieved through the primary database searches were imported into a reference-management system. Duplicate records were removed before title and abstract screening. Potentially eligible articles then underwent full-text review according to the predefined inclusion and exclusion criteria described in the Methods section of the manuscript.

No language restrictions were applied during the initial database search. Database-specific syntax, field labels, and controlled vocabularies were adapted to the requirements of each search platform.

Statement on Reproducibility

The search strategies presented in this appendix document the database-specific search structure used during the review. Syntax, field labels, and controlled vocabularies were adapted to the requirements of PubMed/MEDLINE, Embase, and IEEE Xplore while preserving the same core conceptual structure. The reported date limits correspond to the search period used in the review.
